# Disparities in telehealth utilization in patients with pain during COVID-19

**DOI:** 10.1097/PR9.0000000000001001

**Published:** 2022-04-14

**Authors:** Bridget R. Mueller, Steven Lawrence, Emma Benn, Sharon Nirenberg, Benjamin Kummer, Nathalie Jette, Mary-Catherine George, Jessica Robinson-Papp

**Affiliations:** Departments of a Neurology and; b Population Health Sciences and Policy, Icahn School of Medicine at Mount Sinai, New York, NY, USA; c Scientific Computing and Data, Icahn School of Medicine at Mount Sinai, New York, NY, USA; d Clinical Informatics, Mount Sinai Health System, New York, NY, USA; e Neurology and Population Health Science & Policy, Icahn School of Medicine at Mount Sinai, New York, NY, USA

**Keywords:** Telehealth, Disparities, COVID-19

## Abstract

**Introduction:**

The shift from in-person visits to telehealth visits during the COVID-19 pandemic presented unique challenges for patients with pain. Disparities in health care access already existed, and the impact of telehealth on these inequities has not been studied.

**Objectives:**

To identify sociodemographic characteristics of patients with pain obtaining care through video, telephone, and in-person visits as social distancing restrictions evolved during the COVID-19 pandemic.

**Methods:**

Using our institutional clinical data warehouse, we identified 3314 patients with pain receiving care at a large academic institution in New York City during a baseline period (September 23, 2019–March 22, 2020) and counted telephone, video, and in-person visits during the following conditions: a shutdown period (March 23, 2020–May 23, 2020), when nonessential in-person visits were strictly limited, and a reopening period (May 23, 2020–September 23, 2020), when restrictions were relaxed and in-person visits were available. Patients were categorized into 4 groups based on the technology used to complete a visit: (1) video, (2) telephone, (3) in-person, and (4) no visit.

**Results:**

Patients who were older, publicly insured, and identified as Black or Hispanic were overrepresented in the telephone visit group during shutdown and the in-person group during reopening. A video visit during shutdown increased the likelihood of continued video visit use during reopening despite the return of in-person visits.

**Conclusions:**

Results show differences in how patients with pain accessed clinical care in a socially distanced world and that flexibility in method of health care delivery may reduce barriers to access. Future research will identify factors (eg, Internet access, digital literacy, provider–patient relationships) driving heterogeneity in telehealth use in patients with pain.

## Abstract 2

Supplemental Digital Content is Available in the Text.

For patients living with pain in New York City during the first wave of COVID-19, the relationship between sociodemographic factors and telehealth utilization evolved with time.

## 1. Introduction

The passage of the Coronavirus (COVID-19) Preparedness and Response Supplemental Appropriations Act in March of 2020 removed barriers that had previously hindered widespread use of telehealth.^41^ Telehealth, defined as clinical care provided through audio-only (telephone) or visual-audio (video) technologies, permitted clinicians to care for patients despite shelter-in-place orders and ushered in a new era for health care delivery.^4,13^ Although 8% of Americans used telehealth in 2019,^43^ 70% of ambulatory visits used telehealth at some institutions by April 2020.^12,29,39^

Unfortunately, disparities in Internet access and health literacy may impede equitable use of telehealth.^8^ This digital divide disproportionately affects racial and ethnic minority groups, those with lower socioeconomic status, and older patients.^3,26,36,41^ Recent studies report inconsistent relationships between sociodemographic factors and telehealth.^7,21^ The medical needs of the study cohort,^12^ social distancing recommendations during the study period, and definition of telehealth (eg, video and audio vs audio only) are important sources of study variability.

Owing to the biopsychosocial complexity of pain disorders, an increased reliance on telehealth may pose specific challenges for patients with pain. Maintaining a therapeutic relationship may be challenging during a telehealth visit, and feelings of loneliness are associated with worsening pain.^35^ Interventional analgesic procedures have helped many patients with pain but require an in-person visit. Finally, studies in patients with pain have shown that the same patient characteristics associated with a low digital access (ie, being older in age, a racial and ethnic minority, and having a lower socioeconomic status) are associated with greater pain severity.^17,23,31,39^ Therefore, patients with severe pain may have the most difficulty connecting with their providers.

Major metropolitan areas on the East Coast of the United States were among the first regions to have high burdens of COVID-19 and therefore served as testing grounds for rapid telehealth implementation. Little is known about how diverse patients with pain used telehealth then. Using a de-identified institutional data warehouse, we identified a cohort of patients with pain receiving care in New York City (NYC) before the pandemic and examined utilization of telephone, video, and in-person visits during 2 periods: (1) when all nonurgent ambulatory visits were conducted by telehealth (March 23, 2020–May 23, 2020) and (2) when COVID-19 infection rates fell and in-person ambulatory visits were again available (May 24, 2020–September 23, 2020). We hypothesized that racial and ethnic minority groups, the elderly individuals, and those with public insurance will have decreased utilization of video compared with that of telephone during shutdown and that these groups will favor in-person visits when they become available during reopening. By describing the sociodemographic characteristics of patients with pain receiving telehealth and in-person care during both time, we aim to provide foundational information that can inform strategies aimed at mitigating access disparities for patients with pain in the post–COVID-19 era.

## 2. Methods

### 2.1. Study design/setting

We conducted a retrospective cohort study at the Mount Sinai Health System (MSHS), a large academic health care system, composed of 7 hospitals in NYC, which uses a single electronic health record (EHR) system (Epic, Verona, WI). Before the pandemic, our institution maintained an infrastructure for telemedicine that expanded rapidly during early March 2020 to offer telehealth at all hospitals and ambulatory sites.^25^ Mount Sinai Health System is a Specialized Clinical Center in the NIH's Helping to End Addiction Long-term (HEAL)-funded Early Phase Pain Investigation Clinical Network (EPPIC-Net) and treats a large population of diverse urban patients with pain. Thirty-three pain specialists from diverse disciplines (eg, anesthesiology, internal medicine, neurology, and psychiatry) across the health system serve as EPPIC-Net providers and a source of referrals for clinical pain trials. These EPPIC-Net providers have pain practices at our Upper East Side, Upper West Side, Union Square, Queens, and Brooklyn locations. All sites had similar protocols for scheduling in-person and telehealth visits during the shutdown and reopening periods. During shutdown, only urgent in-person evaluations were scheduled, and patients were encouraged to see their provider using a video platform. If technological limitations prevented a video visit or the patient had a preference for audio-only communication, a telephone visit was conducted. There was no difference in cost of in-person, telephone, and video visits to the patient. Owing to policies enacted by the Centers for Medicare and Medicaid (CMS), reimbursement for telehealth visits was equivalent to in-person office visits.^41^

### 2.2. Study sample

We extracted a de-identified data set of eligible patients from the Mount Sinai Data Warehouse (MSDW) using the following inclusion criteria: (1) at least 1 office visit with an MSHS EPPIC-Net pain provider in the 6-month baseline period (September 24, 2019–March 22, 2020) and (2) at least 1 pain diagnosis used to bill a visit during this same baseline period. Pain diagnoses were identified using a previously published list of *International Classification of Disease (ICD)-9* codes^24^ and translated to *ICD-10* codes (see Supplemental Material for complete list, available at http://links.lww.com/PR9/A156). The Mount Sinai Hospital Institutional Review Board approved the use of patient data for this retrospective study and waived the requirement for informed consent.

### 2.3. Data collection and characterization of study sample

We collected baseline patient characteristics that included race and ethnicity, age, sex, primary health insurance category (Medicaid, Medicare, or private insurance), office visit billing pain diagnosis, number of MSHS providers seen during baseline, and zip code of patients' billing address. Patients self-report sex, race, and ethnicity at their initial Mount Sinai appointment in a demographic intake form, and this information is saved in the EMR. If a patient's race and ethnicity is not listed on the patient demographic intake form, the patient may self-report other or write in their specific race and ethnicity. Race and ethnicity cohorts comprising less than 0.5% of the sample population are categorized as other. Patients who choose not to self-report race and ethnicity are categorized as not specified. Our race and ethnicity categorization is based on recent guidelines published by the Journal of the American Medical Association (JAMA)^14^ and the Mount Sinai office for Diversity and Inclusion.^46^ Zip code of patients' billing address was categorized as NYC or non-NYC.

#### 2.3.1. Outcomes

We defined 2 periods: shutdown (March 23, 2020–May 23, 2020) and reopening (May 24, 2020–September 23, 2020). The shutdown period encompasses the time when nonurgent in-person office visits at MSHS were prohibited. Only rarely, when an in-person physical examination was necessary and an ED visit was not appropriate, a provider could arrange for an in-person ambulatory visit. The reopening period encompasses the time during which restrictions were relaxed and in-person office visits were allowed. The data warehouse was queried for all outpatient visit encounters using billing codes submitted by the MSHS provider after visit completion. For each patient with pain in the study cohort, we counted in-person, video, and telephone visits with all MSHS providers during shutdown and reopening periods. Incomplete and no-show visits were not included in the data set. Our data set excluded in-patient hospitalizations, urgent care, and ED visits.

Patients were grouped according to technology used to complete a visit during each period (shutdown and reopening). The video visit group included patients with at least 1 video visit, regardless of whether they also had a telephone or in-person visit. The telephone visit group included patients with at least 1 telephone visit, but no video visits, regardless of in-person visits. Patients in the in-person visit group did not engage in telephone or video visits and had at least 1 in-person ambulatory visit. The remainder of the patients were in the no visit group. Visit group categorization was determined separately for shutdown and reopening periods. For example, a patient could be in the telephone group during shutdown and video group during reopening. Our primary outcome was visit group during shutdown and reopening periods.

### 2.4. Statistical analyses

Descriptive statistics were first performed for the clinical and demographic variables during shutdown and reopening periods. Patients' age, sex, race and ethnicity, and insurance type and number of baseline providers were compared between the visit groups. We used analysis of variance (ANOVA) or Mann-Whitney *U* test to compare continuous variables and the χ^2^ test to compare categorical variables, respectively, between visit groups.

Binomial logistic regression identified patient characteristics independently associated with the video vs telephone visit groups during shutdown. During reopening, multinomial logistic regression was used to determine patient characteristics independently associated with telephone, video, and in-person visit groups, as well as assess the influence of the shutdown telehealth experience on reopening care. Because in-person care during shutdown likely represented an urgent need and there were relatively few such visits, the in-person group was not included in the shutdown regression. Small sample size precluded inclusion of the Asian and other race and ethnicity groups. The not specified group was not included in the model because of heterogeneity. The zip code variable was omitted from analysis because of collinearity with race. All analyses were performed using R 4.03 (R Foundation for Statistical Computing, Vienna, Austria). A 2-tailed significance level of <0.05 was used for all analyses and controlled for multiple comparisons using the Holms method.

## 3. Results

### 3.1. Sample characteristics

Table 1 summarizes characteristics of our final study sample. We identified 3314 patients meeting inclusion criteria, of which 2330 (70.3%) resided in NYC. Our study sample was diverse. Regarding race and ethnicity, 15.1% self-reported race and ethnicity as non-White and Hispanic (hereafter, Hispanic), 14.4% as Black and African American (hereafter, Black), 42.3% as White, and 4.2% as Asian. The other group (7.6%) was composed of 15 race and ethnicities, each with an N between 1 and 5 (total N = 30), as well as patients who indicated their race and ethnicity as other and did not provide additional details (N = 224). Regarding sex, 37.1% were male individuals and 62.9% were female individuals. Regarding insurance, 13.7% were insured by Medicaid, 29.1% by Medicare, and 57.2% by private insurance. Approximately half (49.6%) of the study cohort carried more than 1 pain diagnosis. The most common pain diagnoses were arthralgia/arthritis (24.9%), back/spinal pain (25.7%), and limb pain (24.4%). A billing diagnosis of chronic pain syndrome was given to 23.1% of patients.

**Table 1:** Characteristics of study cohort.

	N = 3314
Age, mean ± SD	54.0 ± 16.2
Patients residing in New York City	2330 (70.3)
Male sex	1231 (37.1)
Race and ethnicity	
Asian	139 (4.2)
Black	476 (14.4)
Hispanic	500 (15.1)
White	1402 (42.3)
Other	254 (7.6)
Not specified	544 (16.4)
Insurance	
Medicaid	455 (13.7)
Medicare	963 (29.1)
Private	1892 (57.2)
Clinical characteristics	
Providers during baseline*	1.0 (1.0, 2.0)
No. of pain diagnoses per patient*	1.0 (1.0, 2.0)
Chronic pain syndrome	764 (23.1)
Neuropathy	202 (6.1)
Headache	274 (8.3)
Arthralgia/arthritis	826 (24.9)
Back/spinal pain	851 (25.7)
Limb pain	809 (24.4)
Other pain syndrome	480 (14.5)

Values are expressed as N (%), unless otherwise indicated.

* Values are expressed as median (interquartile range).

### 3.2. Visit groups during shutdown

In the following section, we use descriptive analyses (Table 2) to report patient characteristics of shutdown visit groups and regression (Table 3) to identify independent factors associated with shutdown video vs telephone groups. During the shutdown period, 584 (17.6%) patients in the study cohort had at least 1 video, telephone, or in-person visit, and 2730 patients (82.4%) did not have a visit documented in the EHR. Age (*P* < 0.001), sex (*P* < 0.001), race and ethnicity (*P* < 0.001), and insurance (*P* < 0.001) significantly differed between the visit groups.

**Table 2:** Shutdown: characteristics of patients in video, telephone, in-person, and no visit groups.

	Video (n = 387)	Telephone (n = 148)	In-person (n = 49)	No visit (n = 2730)	*P*
Age, y, mean ± SD	53.6 ± 14.9	61.2 ± 13.6	62.3 ± 13.1	53.5 ± 16.5	<0.001
Sex					<0.001
Female	251 (12.0)	115 (5.5)	27 (1.3)	1690 (81.1)	
Male	136 (11.0)	33 (2.7)	22 (1.8)	1040 (84.5)	
Race and ethnicity					<0.001
Asian	20 (14.4)	2 (1.4)	0 (0.0)	117 (84.2)	
Black	57 (12.0)	44 (9.2)	9 (1.9)	366 (76.9)	
Hispanic	62 (12.4)	43 (8.6)	17 (3.4)	378 (75.6)	
White	198 (14.1)	34 (2.4)	18 (1.3)	1152 (82.2)	
Other	25 (9.8)	15 (5.9)	2 (0.8)	212 (83.5)	
Not specified	25 (4.6)	10 (1.8)	3 (0.6)	505 (93.0)	
Insurance					<0.001
Medicaid	45 (9.9)	35 (7.7)	10 (2.2)	365 (80.2)	
Medicare	112 (11.6)	74 (7.7)	27 (2.8)	750 (77.9)	
Private	230 (12.2)	39 (2.1)	12 (0.6)	1611 (85.1)	

Values are as N (% row), unless otherwise indicated.

**Table 3:** Shutdown: binomial logistic regression examining predictors of video visit vs telephone visit.

	Crude video vs telephone OR (95% CI)	Crude *P*	Adjusted video vs telephone OR (aOR) (95% CI)	Adjusted *P*
Age	0.96 (0.95, 0.98)	<0.001	0.96 (0.94, 0.98)	<0.001
Male sex	1.89 (1.23, 2.96)	0.004	1.59 (0.99, 2.62)	0.058
Baseline providers	0.99 (0.96, 1.02)	0.393	1.01 (0.98, 1.04	0.685
Race and ethnicity (reference: White)		<0.001		<0.001
Black	0.22 (0.13, 0.28)		0.27 (0.15, 0.48)	
Hispanic	0.25 (0.14, 0.42)		0.31 (0.17, 0.57)	
Insurance (reference: Private)		<0.001		<0.001
Medicaid	0.22 (0.12, 0.38)		0.29 (0.16, 0.54)	
Medicare	0.26 (0.16, 0.40)		0.50 (0.29, 0.86)	

#### 3.2.1. No visit group

Patients in the no-visit group were younger than those in the telephone group (mean 53.5 years vs 61.2 years) and in-person group (mean 62.3 years) and similar in age to the video group (mean 53.6 years). Relatively more male individuals (85.4%) than female individuals (81.1%) had no visit during shutdown. Compared with Black (76.9%) and Hispanic (75.6%) patients, relatively more White (82.2%) and Asian (84.2%) patients had no visit during shutdown. More patients with private insurance (85.1%) had no visit during shutdown compared with those with Medicaid (77.9%) and Medicare (80.2%).

#### 3.2.2. Telehealth groups (video and telephone)

Two-thirds of the patients who received medical care during shutdown did so using video (N = 387/584, 66.2%), and approximately one-quarter did so using telephone (N = 148/584, 25%). Race and ethnicity was a significant independent predictor of video vs telephone groups (*P* < 0.001, see Table 3 for parameter estimates). For patients engaged in telehealth, 85% of Whites used video, compared with 56% of Black patients and 59% of Hispanic patients. Insurance was also a significant independent predictor of video visits (*P* < 0.001). Among patients engaged in telehealth, 86% of those with private insurance used video compared with 56.2% of patients with Medicaid and 60.2% of patients with Medicare. Patients in the video visit group were younger than the patients in the telephone group (mean 53.6 years vs 61.2 years), and age was a significant independent predictor of video vs telephone group (*P* < 0.001). For patients engaged in telehealth, 80.5% of male individuals and 68.6% of female individuals were in the video group, and there was a trending but insignificant independent effect of sex on video visit group in regression (*P* = 0.058).

#### 3.2.3. In-person visit group

During shutdown, very few patients had in-person visits (N = 49). The in-person group had a similar demographic profile to the telephone group. On average, patients in the in-person group were older than patients in the video-visit group and had relatively more Black and Hispanic patients and patients with public forms of insurance (Table 2).

### 3.3. Visit groups during reopening

Compared with shutdown, the number of patients receiving care during reopening more than doubled (from 1217 to 3314). This increase stemmed from growth of the in-person group (N = 689), which had a greater representation of Black, Hispanic, and publicly insured patients. As was seen during shutdown, a greater proportion of Black, Hispanic, and publicly insured patients received care (of any visit type) during reopening, and age (*P* < 0.001), sex (*P* < 0.001), race and ethnicity (*P* < 0.001), and insurance (*P* < 0.001) differed between the visit groups (Table 4). In the video visit group, the average patient age, percentage of minorities, and ratio of public/private insurance remained similar compared with shutdown (ie, younger, with relatively fewer Black and Hispanic patients, and those with public insurance). Patients in the telephone visit group during reopening were also similar to those in the shutdown period (ie, older, with a greater representation of Black and Hispanic patients, and those with public insurance).

**Table 4:** Reopening: characteristics of pain patients in video, telephone, in-person, and no visit groups.*

	Video (n = 404)	Telephone (n = 135)	In-person (n = 678)	No visit (n = 2097)	*P*
Age, y, mean ± SD	53.9 ± 14.9	60.4 ± 13.6	58.3 ± 14.0	52.2 ± 16.8	<0.001
Sex					<0.001
Female	270 (13.0)	99 (4.8)	456 (21.9)	1258 (60.4)	
Male	134 (10.9)	36 (2.9)	222 (18.0)	839 (68.2)	
Race/ethnicity					<0.001
Asian	18 (12.9)	3 (2.2)	29 (20.9)	89 (64.0)	
Black	59 (12.4)	37 (7.8)	139 (29.2)	241 (50.6)	
Hispanic	61 (12.2)	35 (7.0)	175 (35.0)	229 (45.8)	
White	195 (13.9)	34 (2.4)	253 (18.0)	920 (65.6)	
Other	29 (11.4)	16 (6.3)	44 (17.3)	165 (65.0)	
Not specified	42 (7.7)	10 (1.8)	38 (7.0)	453 (83.4)	
Insurance					<0.001
Medicaid	57 (12.5)	28 (6.2)	141 (31.0)	229 (50.3)	
Medicare	131 (13.6)	63 (6.5)	251 (26.1)	518 (53.8)	
Private	216 (11.4)	44 (2.3)	283 (15.0)	1179 (71.7)	

* Values are as N (% row), unless otherwise indicated.

**Figure 1.**
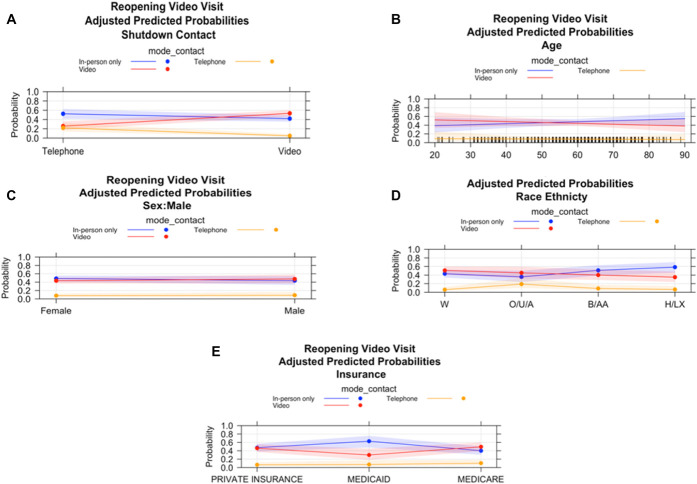
Predicted probabilities of visit group types based on multinomial regression. See Table 5 for full parameter estimates. B/AA, Black/African American; H/LX, Hispanic/LatinX; W, White.

A multinomial regression identified patient factors independently associated with reopening visit group and assessed how telehealth experience during shutdown influenced the use of telehealth during reopening, when routine in-person visits were again an option. Figure 1 is derived from the multinomial regression (see Table 5 for point parameter estimates) and shows that sex (Fig. 1A), age (Fig. 1B), and insurance (Fig. 1C) did not independently predict visit group during reopening. In unadjusted analyses, Black and Hispanic patients were underrepresented in the video group compared with in-person visits, but a Black or Hispanic race was not an independent predictor of video visits during reopening. Experience with telehealth during shutdown was a dominant driver of reopening visit group type (Fig. 1E). Patients in the video group during shutdown were more than twice as likely as patients in the shutdown telephone group to continue using video compared with in-person visits during reopening (aOR = 2.59, 95% CI: 1.44–4.66).

**Table 5:** Reopening: multinomial logistic regression examining predictors of video vs telephone vs in-person groups.

Effect	Visit group	Crude OR (CI)	Crude *P*	Adjusted OR (CI)	Adjusted *P*
Age	Video vs in-person	0.99 (0.98, 1.01)	0.311	0.99 (0.97, 1.01)	0.644
Age	Telephone vs in-person	1.01 (0.99, 1.04)	0.311	0.99 (0.95, 1.02)	0.506
Male	Video vs in-person	1.56 (0.95, 2.55)	0.143	1.26 (0.74, 2.17)	0.562
Male	Telephone vs in-person	0.90 (0.39, 2.07)	0.143	1.48 (0.61, 3.55)	0.562
Shutdown video group	Video vs in-person	3.17 (1.84, 5.48)	<0.001	2.59 (1.44, 4.66)	<0.001
Shutdown video group	Telephone vs in-person	0.29 (0.13, 0.62)	<0.001	0.27 (0.12, 0.63)	<0.001
Baseline providers	Video vs in-person	1.02 (0.99, 1.06)	0.285	1.04 (1.00, 1.09)	0.051
Baseline providers	Telephone vs in-person	1.03 (0.98, 1.07)	0.211	1.05 (0.99, 1.10)	0.052
Race and ethnicity (ref: White)			<0.001		0.064
Black	Video vs in-person	0.47 (0.26, 0.87)		0.67 (0.34, 1.31)	
Black	Telephone vs in-person	2.03 (0.75, 5.51)		1.37 (0.46, 4.13)	
Hispanic	Video vs in-person	0.36 (0.20, 0.68)		0.51 (0.25, 1.02)	
Hispanic	Telephone vs in-person	1.20 (0.40, 3.56)		0.89 (0.27, 2.94)	
Insurance (ref: Private)			0.004		0.167
Medicaid	Video vs in-person	0.36 (0.18, 0.71)		0.49 (0.23, 1.04)	
Medicaid	Telephone vs in-person	0.95 (0.33, 2.80)		0.75 (0.23, 2.48)	
Medicare	Video vs in-person	0.89 (0.54, 1.47)		1.27 (0.68, 2.37)	
Medicare	Telephone vs in-person	2.23 (0.99, 5.03)		1.04 (1.00, 1.09)	

## 4. Discussion

In this retrospective cohort study, we examined utilization of telehealth and in-person visits by patients living with pain at an academic center in NYC during the first wave of COVID-19. This study is the first to focus on patients living with pain and examine how telehealth use evolved as in-person visits became available. We report several novel findings. First, patients with pain who were older, Black, Hispanic, and publicly insured had an increased likelihood of accessing medical care (of any visit type) during both shutdown and reopening periods. Second, during the shutdown of nonurgent in-person visits, these patients were more likely to obtain care through telephone, not video, whereas during reopening, in-person visits predominated, and disparities in video visit use were mitigated. Finally, a video visit during shutdown was a strong independent predictor of continued video use after in-person visits returned.

In our population of patients with pain, we found that a greater proportion of patients who are Black and Hispanic received care during shutdown and reopening periods. This result was not expected and, to the best of our knowledge, not previously reported. Several reasons may account for this pattern. Previous work has demonstrated that patients of minority race and ethnicity and those of lower socioeconomic status are more likely to experience severe pain,^31,39^ which may increase the likelihood of seeking care. The biopsychosocial model of pain also highlights the important contribution of stress in modulating the individual pain experience. The differential impact of COVID-19 on minorities and those with a lower socioeconomic status^45^ may have led to exacerbations of pain, increasing the likelihood of a medical visit.^1,28^ Finally, chronic medical conditions that are prevalent in people who are Black or Hispanic may have also an increased need for medical care.^16,22^ The female predominance in our study population is consistent with previous work demonstrating an increased pain prevalence among female individuals relative to male individuals.^19,32,33,41^ We found a greater utilization of medical care by female pain patients during both shutdown and reopening. This finding is consistent with previous research showing male individuals may underutilize healthcare services, a pattern that may be exacerbated by the stigma of pain.^30,34^

Previous research examining sociodemographic factors and use of telehealth demonstrate a context-dependent relationship. For example, at a Midwest academic center, family medicine telehealth visits were used less often by racial and ethnic minority groups.^37^ At a Northeast academic institution, patients who identified as Asian showed lower utilization than patients who identified as Black or White.^42^ However, in a subgroup analysis comparing telephone and video visits, patients identified as Black were less likely to complete a video visit than those who identified as White.^42^ Our study supports the importance of distinguishing between telephone and video visits.^21,47^ During shutdown, older patients, patients identified as Black and Hispanic, and publicly insured patients were significantly more likely to use telephone vs video than younger, White, privately insured patients. Although this study does not establish the causes underlying these patterns of health care utilization, several hypotheses can be explored in future work. Known disparities in broadband access and technology literacy may contribute to these patterns.^9^ In addition, people of lower socioeconomic status may have reduced access to a private space at work and home. An audio-only telehealth visit might afford greater privacy or flexibility and therefore be preferable for some patients.^38^

High utilization of telephone communication may have important consequences on health outcomes. Despite the establishment of parity for telephone visits by the Centers for Medicare and Medicaid Services (CMS), telephone visits are associated with lower patient satisfaction and inferior communication of medical information in comparison with video visits.^20,27,44^ For patients with limited English proficiency, telephone visits are especially challenging.^27^ Visual information may be particularly important for providers caring for pain patients because nonverbal cues can provide insight into the individual pain experience. Qualitative methods may allow a more nuanced understanding of the experiences felt by patients with pain during the COVID-19 pandemic when in-person visits were not an option.

Our findings provide additional insight beyond previously published studies by examining how disparities in telehealth evolved with time. During reopening, the disparity in telephone vs video visit use among patients with pain who were publicly insured and identified as Black and Hispanic was mitigated and among older patients abolished. This is likely due to the development and implementation of outreach efforts that took time to execute and included assisting patients with portal activation and offering video visits through multiple platforms. Previous work has demonstrated positive effects of such outreach efforts.^18^ The strong relationship between video visit group during shutdown and continued video use during reopening aligns with the popularity of video visits and their continued availability despite a decreased need to social distance due to declining rates of COVID-19 infection. For patients with pain that limits mobility, TH may offer a significant benefit.

Several reasons may account for disproportionate representation of patients who identify as Black or Hispanic in the in-person group during reopening. The importance of an in-person contact may have outweighed a potential increased risk of COVID-19 exposure. Historical mistreatment and current systemic inequalities may contribute to a wariness of technological innovations by the medical community. In addition, there is evidence that face-to-face contact may play a more important role in therapeutic alliance and rapport building for race and ethnic minority individuals.^15,43^

Our study had several limitations. First, our data lacked demographic characteristics associated with digital literacy such as level of education, preferred language, and income. Our data also lacked visit satisfaction and pain severity scores because this information was not uniformly collected. Second, our data are from a single large health system and patients could have had encounters at a different health system. Third, our urban study population may limit the study's generalizability to rural areas where broadband Internet is not as readily available. Fourth, due to the large number of medical specialties represented by our 33 EPIC-Net pain providers, a formal comparison of patients across provider disciplines would be confounded by individual provider practices. Finally, because almost all patients of Black and Hispanic race and ethnicity resided in NYC, the influence of a patient's geographic residence on telehealth utilization could not be examined.

In summary, our study characterizes how patients with pain used telephone, video, and in-person visits during the COVID-19 pandemic and describes how telehealth use patterns evolved as social distancing restrictions were relaxed and in-person visits became available. This important information can be used to guide the formation and implementation of inclusive and flexible telehealth services and policies to prevent widening of existing disparities for patients with pain. Future qualitative studies are needed to understand barriers and potential solutions to adoption of telehealth by patients with underrepresented pain.

## Disclosures

The authors have no conflict of interest to declare.

This work was submitted as a poster and presented orally at the HEAL annual meeting from May 17, 2021, to May 19, 2021.

## Appendix A. Supplemental digital content

Supplemental digital content associated with this article can be found online at http://links.lww.com/PR9/A156.

## Supplementary Material

SUPPLEMENTARY MATERIAL
