# Supplementary material for: Disparities in telehealth utilization in patients with pain during COVID-19
Source: Pain Rep. 2022 Apr 14;7(3):e1001. doi: 10.1097/PR9.0000000000001001 (PMC9015206; doi:10.1097/PR9.0000000000001001)
Supplement: SUPPLEMENTARY MATERIAL [file painreports-7-e1001-s001.pdf]

Supplemental Material

ICD10 codes for pain:

M05.642', 'S12.101B', 'S12.111D', 'S12.130A', 'S12.650D', 'G44.321', 'M16.52', 'M19.222', 'M12.531', 'M1A.4120', 'M86.619', 'M25.769', 'S22.002B', 'S22.049D', 'M06.332', 'S12.121D', 'S12.350S', 'S32.002K', 'S32.129B', 'F45.42', 'S22.069K', 'M05.372', 'M60.851', 'S32.131K', 'S34.132D', 'M25.562', 'S44.01XA', 'M05.331', 'M05.379', 'M05.632', 'M06.211', 'M49.83', 'S14.104S', 'S12.24XG', 'S12.431G', 'S14.126S', 'S14.155D', 'S32.011S', 'S32.012K', 'S34.101A', 'M10.032', 'M86.571', 'M77.10', 'S44.02XS', 'E08.40', 'M05.512', 'M99.57', 'S24.152D', 'S24.154D', 'M05.719', 'S12.040K', 'S12.100B', 'S12.530K', 'S32.028A', 'S32.042G', 'S32.130K', 'M17.0', 'M19.221', 'M19.272', 'M00.262', 'M02.341', 'M25.529', 'S22.032D', 'M06.00', 'M12.041', 'M87.251', 'S12.091A', 'S12.451B', 'S12.691S', 'G44.84', 'S32.000G', 'M15.9', 'M00.119', 'M00.219', 'G57.30', 'S22.078B', 'S22.081K', 'J34.9', 'S12.34XS', 'S12.501A', 'S12.551K', 'S32.019S', 'G43.401', 'G43.A0', 'M62.432', 'M12.552', 'M1A.0391', 'M1A.0410', 'M1A.20X0', 'M1A.3210', 'M1A.3590', 'M86.38', 'M99.54', 'S22.081D', 'S22.24XK', 'M06.1', 'M05.862', 'M32.8', 'S32.119S', 'S32.129A', 'M10.152', 'M1A.0620', 'M86.352', 'M54.13', 'S22.029G', 'S24.113S', 'S22.20XD', 'M06.28', 'S14.114S', 'S14.135D', 'G56.43', 'G44.229', 'S32.040G', 'S32.041D', 'M90.812', 'M70.42', 'G65.1', 'M05.549', 'S22.000K', 'S22.000S', 'S22.23XK', 'S22.41XK', 'T14.8XXD', 'M05.229', 'M46.88', 'S14.123A', 'S12.450D', 'S12.601G', 'M05.042', 'M12.031', 'S12.041A', 'S12.121G', 'S12.450K', 'S12.531D', 'G56.42', 'G44.51', 'M12.532', 'M1A.0611', 'M1A.09X0', 'M1A.4490', 'M86.541', 'E13.42', 'M05.20', 'M50.822', 'S12.031B', 'S12.110G', 'S12.491G', 'S14.155S', 'S32.040S', 'S32.041A', 'M00.072', 'M10.141', 'M1A.38X1', 'G57.22', 'G57.62', 'G57.91', 'S24.101D', 'M05.049', 'M06.271', 'M50.120', 'S12.130K', 'S32.131A', 'M10.28', 'M1A.2290', 'M86.651', 'M86.379', 'M90.80', 'M46.80', 'S12.591D', 'S32.010G', 'S32.018K', 'S32.031K', 'M00.89', 'M01.X19', 'M1A.2590', 'M1A.3510', 'M1A.3711', 'M1A.3720', 'M1A.4420', 'M26.623', 'M86.421', 'S54.01XS', 'E11.40', 'G56.20', 'S24.103S', 'M05.219', 'M05.522', 'M05.531', 'M05.549', 'M12.019', 'S12.041D', 'S12.251D', 'S14.106D', 'G44.221', 'M62.471', 'M12.50', 'S22.002D', 'S24.112A', 'M05.629', 'M05.771', 'S14.152S', 'S12.34XG', 'S12.350K', 'M99.63', 'S32.050D', 'M62.451', 'M00.142', 'M01.X31', 'M02.212', 'M1A.3220', 'M1A.3691', 'M86.529', 'M86.472', 'M86.48', 'M89.671', 'M90.811', 'S64.02XS', 'S22.052A', 'M05.532', 'M06.051', 'M06.372', 'M50.221', 'M46.43', 'S12.350A', 'S12.390B', 'S12.430K', 'S14.156A', 'S12.531B', 'S12.630K', 'M54.40', 'S32.052D', 'M00.111', 'M02.179', 'M12.529', 'M67.369', 'M66.212', 'M75.81', 'M76.70', 'M99.58', 'S32.009D', 'S32.2XXS', 'S22.022B', 'S22.079G', 'M06.319', 'M46.83', 'S12.64XK', 'M26.643', 'M54.5', 'M10.071', 'M1A.4711', 'M76.811', 'G56.90', 'S22.038K', 'M05.041', 'M05.479', 'M06.242', 'S32.010K', 'S32.052S', 'S34.104S', 'S32.121S', 'M00.862', 'M1A.0190', 'G61.1', 'M99.25', 'M99.55', 'S22.009A', 'M05.061', 'M50.00', 'S12.301A', 'M26.641', 'S32.112G', 'S32.15XD', 'M62.411', 'M00.271', 'M1A.2110', 'M86.519', 'M65.129', 'S22.070D', 'M05.752', 'M06.212', 'M87.011', 'S12.190G', 'S32.012B', 'S32.059B', 'G43.811', 'M76.00', 'G56.23', 'S22.021A', 'M05.369', 'M05.849', 'M50.90', 'S12.200D', 'S12.400K', 'S32.119K', 'S34.132S', 'M10.151', 'S54.01XD', 'S22.008A', 'S22.069S', 'S22.078A', 'M50.922', 'S12.230B', 'S12.550B', 'S12.690D', 'S32.011B', 'S32.041G', 'M19.212', 'M02.332', 'M25.50', 'E11.42', 'M05.522', 'M48.00', 'S22.039K', 'S12.390D', 'S12.530A', 'S32.022K', 'S32.050K', 'M01.X69', 'M02.879', 'M46.21', 'G58.7', 'S22.068K', 'S22.43XB', 'M12.071', 'M50.220', 'S14.112A', 'S12.34XA', 'S12.400G', 'S12.651A', 'M60.831', 'S32.021A', 'S32.059A', 'M16.9', 'M12.539', 'M89.639', 'M46.09', 'S32.009K', 'S22.062A', 'M05.321', 'S12.251G', 'S12.401D', 'S32.000A', 'S34.119S', 'M02.111', 'M1A.4710', 'M86.372', 'M89.649', 'M65.111', 'S22.042K', 'S22.078K', 'M46.87', 'M87.875', 'S12.201D', 'S32.042B', 'M02.252', 'M86.349', 'M76.01', 'M05.529', 'M99.29', 'S22.24XA', 'M05.269', 'M05.579', 'S12.151S', 'S12.601B', 'M26.632', 'S32.16XD', 'M00.812', 'M1A.2121', 'M90.842', 'I70.232', 'M65.151', 'M65.89', 'M76.21', 'M51.34', 'M05.621', 'M12.012', 'M87.159', 'S12.300B', 'S12.150G', 'S12.690A', 'S32.050S', 'S34.119D', 'S32.112K', 'M00.232', 'M12.59', 'G55', 'G57.93', 'M05.569', 'M99.56', 'S32.009A', 'S22.039B', 'S22.052S',

'S22.079D', 'M30.8', 'M46.85', 'G44.82', 'S32.029G', 'S32.122D', 'M17.31', 'M19.029', 'M18.52',
'M00.19', 'M90.829', 'M67.312', 'M99.68', 'S32.009S', 'S22.030D', 'M06.339', 'M87.276', 'S12.110A',
'S12.111G', 'S12.231A', 'S32.029S', 'S32.048D', 'S34.103D', 'M19.249', 'M02.142', 'M10.231',
'S22.030A', 'S22.088D', 'S22.089G', 'M05.032', 'M06.09', 'M32.14', 'S12.14XS', 'S32.130A',
'S32.132B', 'M15.4', 'M10.079', 'M10.159', 'M86.641', 'I73.01', 'M46.06', 'S22.011D', 'S24.112S',
'S22.21XG', 'M05.569', 'M87.322', 'S12.301D', 'S14.137A', 'M31.6', 'S32.058G', 'S32.139G',
'M00.239', 'M02.849', 'M1A.4720', 'M1A.4791', 'M90.869', 'M65.161', 'G60.3', 'M46.49', 'S24.109D',
'S22.011G', 'S22.021B', 'S22.021K', 'M05.851', 'S12.331B', 'S12.531G', 'M19.019', 'M19.172',
'M90.832', 'M66.219', 'S22.038S', 'M05.051', 'S14.113A', 'S12.631A', 'S34.115A', 'M00.829',
'M00.832', 'M1A.2310', 'I70.239', 'S22.078D', 'M34.2', 'J34.1', 'S14.132D', 'S14.133D', 'S32.052K',
'S32.17XG', 'M1A.2491', 'I70.243', 'M75.00', 'S22.040G', 'M05.439', 'S32.008K', 'S32.129G',
'S32.16XS', 'M00.052', 'M1A.0390', 'S64.02XD', 'M99.35', 'S22.018K', 'S22.21XS', 'S22.41XA',
'M06.039', 'M06.221', 'M50.920', 'S12.030S', 'S12.190S', 'M00.042', 'M10.132', 'M1A.0721',
'M1A.2690', 'M90.849', 'M79.662', 'G60.9', 'G65.0', 'S22.009G', 'M06.30', 'M87.021', 'M87.821',
'S12.091K', 'S12.24XB', 'S12.500D', 'S12.601D', 'M51.16', 'S32.001K', 'S32.039A', 'M19.132',
'M86.552', 'M54.11', 'M05.69', 'M32.11', 'M50.322', 'S14.112D', 'S12.54XS', 'S12.590G', 'S32.119B',
'M19.131', 'M18.30', 'M19.29', 'M01.X51', 'M02.39', 'M10.019', 'M1A.3310', 'M26.629', 'I70.213',
'M46.00', 'M51.35', 'M05.529', 'M90.50', 'S14.153A', 'S12.400A', 'S12.630D', 'G50.0', 'G43.711',
'M86.361', 'I70.231', 'M65.819', 'S64.01XS', 'G62.82', 'S22.061S', 'M05.7A', 'M06.041', 'M32.13',
'M87.150', 'S12.001G', 'S12.491S', 'S14.117S', 'S34.111A', 'M10.279', 'M1A.4291', 'M05.579',
'M54.18', 'M99.69', 'M05.019', 'G44.009', 'S32.131S', 'M00.841', 'M00.851', 'M86.332', 'M25.762',
'B02.21', 'S22.019B', 'M05.512', 'M87.00', 'S14.123S', 'S12.590D', 'S32.010A', 'S32.042A', 'M62.431',
'M02.352', 'M10.121', 'M1A.3191', 'M77.32', 'B02.0', 'M99.79', 'S22.001A', 'S22.038B', 'S22.060G',
'S22.071K', 'S22.42XS', 'S12.031S', 'S12.390K', 'S14.117D', 'S12.64XG', 'S32.022A', 'S32.058B',
'S34.109A', 'S32.120K', 'M02.221', 'M36.3', 'N25.0', 'M25.761', 'M66.211', 'G61.81', 'M99.66',
'S22.048B', 'S22.061D', 'S22.23XD', 'S22.43XG', 'M05.539', 'M12.09', 'M46.50', 'S14.105A',
'S34.3XXD', 'M02.862', 'M25.549', 'M86.671', 'M54.15', 'M34.89', 'S12.090B', 'M60.822', 'S32.029D',
'S32.132K', 'G43.839', 'M62.459', 'M01.X59', 'M02.329', 'I70.261', 'M76.11', 'S24.102D', 'M05.312',
'M87.122', 'M87.176', 'S14.107S', 'M26.622', 'S32.020K', 'M01.X9', 'M10.051', 'M86.68', 'M25.751',
'M54.89', 'M05.552', 'M06.249', 'S14.154D', 'S12.350G', 'S12.500G', 'M00.012', 'M10.041', 'M12.9',
'M25.541', 'M83.0', 'S22.009S', 'M06.321', 'S12.330G', 'S14.115D', 'S32.030D', 'S32.059S',
'S32.10XD', 'S32.2XXS', 'S34.131S', 'M02.272', 'M1A.2411', 'M90.839', 'I70.219', 'M76.822',
'S22.059B', 'M05.222', 'S34.112D', 'S32.121K', 'G43.601', 'M19.071', 'M10.18', 'M86.8X9', 'M86.329',
'I70.218', 'M67.359', 'M70.50', 'M05.462', 'M87.321', 'S12.031D', 'S12.300G', 'S12.330S', 'S12.530G',
'S32.000B', 'S32.15XA', 'M62.441', 'M02.839', 'M1A.3321', 'M86.549', 'I73.00', 'M70.10', 'M70.60',
'G56.22', 'M99.32', 'S22.32XD', 'M05.361', 'M05.749', 'M06.351', 'M50.23', 'S14.153S', 'S12.131K',
'M48.062', 'M10.261', 'M10.271', 'M25.752', 'S22.012G', 'S22.060A', 'M05.852', 'M46.51', 'M46.95',
'S12.431D', 'G44.091', 'G43.609', 'M18.50', 'M02.371', 'M10.161', 'M90.852', 'M75.100', 'S22.040K',
'S22.058B', 'S22.058S', 'S22.072B', 'S22.082K', 'S22.43XS', 'S12.150K', 'S12.451D', 'S13.4XXS',
'N94.819', 'S34.123S', 'M00.062', 'M01.X32', 'M77.12', 'S44.00XS', 'S54.00XS', 'M05.351', 'M05.611',
'M05.639', 'M05.469', 'M87.275', 'M96.1', 'S12.090K', 'S12.34XD', 'S12.501B', 'S12.691K', 'R10.10',
'M26.601', 'S32.041S', 'M19.92', 'M00.00', 'M1A.4310', 'M89.669', 'M25.741', 'S22.032A', 'S22.062S',
'M05.311', 'S14.132A', 'S12.691A', 'S32.010B', 'S32.049K', 'S32.122K', 'S32.17XK', 'M18.11',
'M02.269', 'M02.359', 'M67.362', 'G57.33', 'S22.22XA', 'M05.541', 'M05.711', 'M46.91', 'M50.323',
'S12.331K', 'S12.351K', 'M17.10', 'M00.241', 'M1A.4321', 'M1A.48X1', 'M26.621', 'M86.58', 'M65.139',
'S22.001D', 'S22.39XS', 'S22.49XS', 'M05.40', 'S12.030G', 'S14.122D', 'S14.136S', 'S12.551D',
'M26.609', 'S32.038D', 'S34.101S', 'M02.231', 'M1A.3320', 'M25.569', 'M46.22', 'I70.262', 'M65.851',
'S22.021S', 'S22.059K', 'S22.089B', 'L93.0', 'M05.29', 'M06.059', 'M75.52', 'M05.572', 'M54.9',
'S32.10XK', 'S22.001S', 'S22.082D', 'M05.821', 'S32.009G', 'S34.129D', 'M1A.4210', 'S44.02XA',
'G57.60', 'S22.030S', 'M06.852', 'M87.175', 'S12.201K', 'S32.000D', 'S32.030B', 'M19.171',
'M10.111', 'M12.541', 'M86.8X8', 'M79.606', 'M99.74', 'S22.082S', 'M06.241', 'M26.619', 'M62.461',
'M19.239', 'M10.029', 'M86.569', 'M65.861', 'M06.062', 'M06.349', 'M12.039', 'M87.876', 'M50.222',
'S12.601A', 'S12.54XB', 'S32.012G', 'S32.19XD', 'M10.012', 'M83.1', 'M65.812', 'G62.1', 'S22.32XA',

'M05.622', 'S12.110S', 'R51.9', 'S32.020G', 'S32.050B', 'G43.001', 'G43.901', 'M00.129', 'M10.211', 'M10.251', 'M1A.4191', 'S22.039A', 'M05.031', 'M05.332', 'M06.849', 'S12.351D', 'S32.17XD', 'M02.131', 'M1A.00X0', 'M1A.4190', 'M86.631', 'M75.02', 'F44.5', 'S22.048D', 'M05.571', 'S12.031A', 'S12.230K', 'S12.350D', 'S32.051A', 'M99.46', 'M99.52', 'S22.048A', 'S22.052B', 'S24.114S', 'S22.31XB', 'M12.029', 'M90.511', 'M50.921', 'S12.331A', 'S12.44XD', 'M99.73', 'M00.212', 'M00.252', 'M10.269', 'M05.551', 'S22.022D', 'S22.081B', 'S22.24XG', 'M05.319', 'M05.741', 'M87.822', 'S12.041G', 'S12.591B', 'M01.X49', 'M36.4', 'M1A.2510', 'G56.91', 'M51.05', 'S22.049B', 'S22.21XK', 'M05.421', 'M87.352', 'M50.10', 'S12.230S', 'M31.5', 'S32.021S', 'S32.039B', 'S34.124D', 'M15.0', 'M10.031', 'M1A.39X1', 'M1A.4511', 'G57.51', 'S34.139D', 'S22.039S', 'S22.080D', 'M05.829', 'S12.110B', 'S12.150B', 'G44.329', 'S32.17XB', 'M00.011', 'M01.X39', 'M46.23', 'M65.152', 'M67.342', 'M70.40', 'M79.676', 'G57.63', 'S22.020K', 'S22.42XB', 'M87.274', 'S12.131S', 'G89.21', 'S32.001A', 'M02.229', 'M1A.4220', 'G56.83', 'M99.45', 'S22.020S', 'S22.39XK', 'M05.261', 'M05.341', 'M05.80', 'S14.157A', 'S12.54XK', 'S32.038K', 'M01.X41', 'M86.559', 'M05.559', 'M51.84', 'S22.009B', 'M05.432', 'M05.442', 'M06.311', 'M30.2', 'M49.86', 'M60.812', 'M60.841', 'S32.111G', 'M16.31', 'M02.88', 'M10.249', 'M72.2', 'S12.090S', 'S32.110S', 'M19.031', 'M00.029', 'M10.232', 'M1A.0420', 'M1A.4411', 'G57.23', 'S22.022A', 'S22.031B', 'M05.241', 'M32.10', 'S14.134D', 'S12.191B', 'M79.11', 'S32.038S', 'S32.049D', 'M1A.3311', 'M90.871', 'M65.179', 'S22.051D', 'S24.151A', 'M06.011', 'M06.839', 'S14.102A', 'S12.54XG', 'S12.691D', 'S34.125D', 'S32.139A', 'S32.19XB', 'S34.3XXS', 'M62.462', 'M16.2', 'M19.049', 'M01.X79', 'M1A.0210', 'M1A.4491', 'M86.8X4', 'M79.632', 'M99.59', 'S14.109D', 'S22.000A', 'S22.012A', 'S22.049S', 'S22.059S', 'S22.080B', 'S12.230G', 'S14.117A', 'S14.137D', 'M79.18', 'M19.90', 'M19.19', 'M02.80', 'M01.X11', 'M1A.3611', 'I70.248', 'M51.37', 'S22.24XB', 'S22.32XK', 'M06.269', 'M46.53', 'M46.59', 'M87.08', 'S12.111B', 'G44.001', 'S32.032D', 'S32.048A', 'M62.838', 'M62.452', 'M1A.3291', 'M86.672', 'M99.24', 'M06.061', 'M06.38', 'M34.83', 'M87.80', 'M87.351', 'S12.400B', 'S32.040A', 'S32.111S', 'M10.179', 'M1A.0510', 'M86.439', 'M25.732', 'E10.40', 'M51.04', 'S22.038A', 'S22.22XG', 'L40.59', 'M05.079', 'M87.222', 'S34.102S', 'M17.32', 'M19.111', 'M00.871', 'M12.542', 'M12.572', 'M79.646', 'M67.351', 'S22.000D', 'M60.811', 'M54.05', 'M19.229', 'M02.121', 'M1A.0690', 'M1A.4221', 'M86.551', 'M67.321', 'M99.65', 'S22.050S', 'S22.058G', 'M05.059', 'M06.042', 'M49.89', 'S12.130G', 'S12.630A', 'S32.048K', 'S34.3XXA', 'M02.372', 'M10.171', 'I70.211', 'B02.29', 'M46.40', 'S12.01XG', 'S14.124D', 'S12.290B', 'S12.401K', 'G44.029', 'S32.000K', 'S32.16XG', 'M18.12', 'M96.1', 'S22.022K', 'S22.042A', 'S12.200K', 'S12.112A', 'S12.130D', 'S12.451G', 'M79.12', 'S32.041K', 'M1A.29X0', 'M99.44', 'S32.10XS', 'M46.90', 'S12.040S', 'S12.64XB', 'G43.919', 'M10.169', 'M1A.2220', 'M89.621', 'M90.872', 'M76.60', 'G62.81', 'S12.9XXS', 'S22.23XA', 'M12.079', 'S12.112D', 'S12.401B', 'S12.64XA', 'M19.91', 'M02.311', 'M86.459', 'M67.372', 'M75.110', 'M77.50', 'M79.675', 'G57.50', 'S12.9XXA', 'S22.039D', 'M06.279', 'M06.80', 'M79.10', 'M51.36', 'M19.129', 'M86.649', 'M86.351', 'M50.821', 'S12.091S', 'S32.132S', 'S32.14XA', 'M16.6', 'M00.259', 'M00.08', 'M10.241', 'M1A.28X0', 'M1A.9XX0', 'S22.39XB', 'S22.49XA', 'M46.42', 'M50.91', 'S12.690K', 'M60.88', 'S32.002G', 'G43.419', 'M1A.30X0', 'G57.20', 'S22.041A', 'S22.070B', 'S32.009B', 'S32.011A', 'S32.051B', 'S34.125A', 'G43.719', 'M54.08', 'M1A.2291', 'M70.70', 'S22.029D', 'S22.050D', 'S22.081G', 'S24.104D', 'M12.042', 'M12.069', 'M87.229', 'S12.030K', 'S12.200S', 'G90.511', 'S32.010S', 'M62.469', 'M19.079', 'M02.19', 'M87.350', 'M50.11', 'S12.450S', 'M86.521', 'M90.851', 'I73.81', 'M75.111', 'M76.52', 'M77.30', 'G57.11', 'S22.42XD', 'M34.1', 'S12.111S', 'S12.34XB', 'S12.491K', 'S32.031B', 'S32.058D', 'M02.20', 'M1A.2410', 'M1A.3211', 'I70.242', 'M46.04', 'S32.2XXA', 'S22.042B', 'M05.729', 'M05.832', 'S14.154S', 'S12.131B', 'S12.390A', 'S12.651G', 'M26.603', 'G44.83', 'S32.112D', 'S32.14XG', 'S32.16XA', 'M16.0', 'M18.9', 'M10.08', 'M11.20', 'M1A.2620', 'M86.59', '70.221', 'F45.8', 'M05.441', 'M87.874', 'S14.134S', 'S12.690G', 'G44.59', 'S32.042S', 'S32.051G', 'M16.4', 'M10.059', 'M1A.0521', 'M89.629', 'S22.050G', 'S22.058K', 'M05.212', 'M06.859', 'M87.221', 'S12.190D', 'S12.401A', 'S12.650K', 'S12.651D', 'R10.30', 'M26.621', 'M19.112', 'M02.829', 'M65.141', 'M76.62', 'S22.040D', 'S24.101A', 'M05.842', 'S12.9XXD', 'G90.513', 'S32.021B', 'M1A.4390', 'M76.51', 'S22.042S', 'S22.052G', 'S12.090D', 'S12.190B', 'S12.550K', 'S12.691G', 'M54.32', 'S32.038G', 'S32.132G', 'M10.052', 'M65.142', 'M75.32', 'E09.42', 'S22.20XA', 'M05.449', 'M05.561', 'S14.116A', 'G44.53', 'S32.048B', 'S34.125S', 'S32.121B', 'M18.31', 'M10.19', 'M86.531', 'M79.646', 'S22.041D', 'M05.751', 'M12.052', 'G56.40', 'M46.47', 'M02.211', 'M50.320', 'S12.530B',

'M60.839', 'M17.4', 'M00.251', 'M86.50', 'I70.212', 'M75.50', 'S44.00XD', 'M99.34', 'M06.361',
'S12.44XB', 'S12.600S', 'G44.019', 'G44.049', 'M48.07', 'S32.042D', 'S32.042K', 'S32.2XXD',
'M00.842', 'M1A.2391', 'M1A.2691', 'M1A.2720', 'M1A.3621', 'M65.862', 'M46.03', 'S22.060K',
'S22.061G', 'S24.134A', 'M06.262', 'S12.44XA', 'S12.490K', 'G44.201', 'M53.88', 'M02.242',
'M89.651', 'M05.541', 'S22.079S', 'M05.859', 'M49.82', 'S12.101D', 'S12.690B', 'S32.19XA',
'M02.842', 'M1A.38X0', 'M1A.4611', 'M83.9', 'M65.121', 'S22.020A', 'S24.153D', 'M87.151', 'M50.83',
'M60.842', 'S32.059D', 'S34.129A', 'M18.2', 'M19.93', 'M86.622', 'M65.171', 'E13.40', 'M51.15',
'S22.43XA', 'M05.232', 'M05.732', 'S32.15XS', 'M01.X71', 'M02.872', 'M10.20', 'M1A.0310', 'M25.59',
'M79.631', 'G57.02', 'S22.029S', 'S24.134D', 'S22.32XG', 'S22.42XA', 'M05.262', 'M05.551', 'M32.15',
'M46.93', 'S12.02XK', 'S12.201S', 'S12.190K', 'S12.431B', 'S32.002A', 'S32.020S', 'G43.019',
'M00.811', 'M86.331', 'M77.02', 'S22.010A', 'S22.41XS', 'M06.022', 'M06.252', 'S12.001S', 'M60.849',
'S32.19XS', 'M00.869', 'M02.822', 'M1A.4721', 'M86.469', 'I70.228', 'G62.9', 'M99.77', 'S22.010B',
'S22.040A', 'S22.069D', 'M87.376', 'S12.040A', 'M26.629', 'S32.010D', 'S32.051K', 'M02.831',
'M1A.0121', 'M86.479', 'M90.89', 'M25.772', 'S22.22XD', 'M06.819', 'M12.072', 'M87.329', 'M87.074',
'S14.152A', 'S32.008G', 'S34.102A', 'S34.113A', 'S32.10XG', 'M65.18', 'M05.519', 'S22.019A',
'S22.071D', 'S22.088A', 'S24.101S', 'S22.23XG', 'S22.23XS', 'M46.98', 'M49.84', 'S12.131D',
'S12.150S', 'S14.116S', 'M53.87', 'M51.86', 'G43.011', 'M17.9', 'M19.149', 'M02.361', 'M10.039',
'M1A.4111', 'M86.8X6', 'M76.10', 'S22.020D', 'S22.030K', 'S22.069G', 'S22.080A', 'S24.114D',
'S22.49XB', 'M05.70', 'M05.811', 'M87.076', 'M53.83', 'S14.122A', 'S12.151D', 'G57.70', 'S32.018B',
'S32.052A', 'M02.139', 'M90.859', 'I70.241', 'M65.821', 'S22.038G', 'S24.111D', 'S22.31XS',
'M06.239', 'M87.029', 'S34.105A', 'G43.611', 'M01.X62', 'M1A.3421', 'M86.312', 'M86.429', 'M89.619',
'M65.4', 'M79.621', 'E08.42', 'S22.062G', 'S22.068S', 'S22.20XS', 'M05.671', 'G44.81', 'M99.23',
'M89.642', 'G57.21', 'S22.049G', 'S12.130S', 'S12.251B', 'S12.291D', 'S12.590K', 'S34.102D',
'S32.16XB', 'M00.88', 'M02.30', 'M1A.30X1', 'M1A.29X1', 'M86.579', 'M70.12', 'S22.060D', 'M05.211',
'M06.031', 'M06.371', 'S12.112B', 'M60.89', 'S32.012A', 'S34.124S', 'G43.501', 'M10.00', 'M1A.2111',
'M1A.2511', 'M51.9', 'S22.031K', 'S22.42XK', 'M05.052', 'M05.329', 'M99.01', 'S12.200G', 'S14.106S',
'R51.0', 'M02.10', 'M1A.3491', 'M46.08', 'M99.37', 'M99.62', 'S22.032B', 'M05.322', 'M87.374',
'S14.114D', 'S32.018G', 'S32.028K', 'S32.049S', 'S32.2XXA', 'S32.139S', 'M1A.3591', 'M25.522',
'M83.5', 'M86.639', 'M65.19', 'F44.7', 'M51.34', 'S22.048S', 'S22.079K', 'S12.300S', 'S12.250G',
'S12.500B', 'G90.529', 'M43.28', 'S32.021G', 'M19.032', 'M02.129', 'M02.171', 'M02.29', 'M65.88',
'M76.30', 'G57.03', 'T14.8XXS', 'M05.09', 'S12.031K', 'S12.200A', 'S14.105S', 'S12.531A',
'S34.121S', 'M15.8', 'M1A.49X1', 'G61.82', 'M54.12', 'S22.31XG', 'M05.872', 'M50.03', 'S12.400D',
'S32.031G', 'M1A.0591', 'M1A.2610', 'M1A.4110', 'M25.571', 'M65.131', 'S22.008S', 'S22.009K',
'S22.082G', 'S22.088K', 'M05.072', 'M05.252', 'M05.649', 'S12.490A', 'S14.125D', 'S12.591K',
'G56.41', 'S32.012S', 'S34.111S', 'S34.114S', 'S32.10XA', 'S32.120S', 'G43.821', 'M16.10', 'M17.2',
'M01.X61', 'M02.119', 'M1A.2211', 'M86.642', 'M65.811', 'G57.01', 'S22.051A', 'M05.039', 'M06.369',
'M06.851', 'M50.022', 'M50.30', 'M50.13', 'S12.01XA', 'S12.100G', 'S12.430D', 'S12.450G',
'S14.156D', 'M26.612', 'M01.X52', 'M1A.2390', 'S54.02XA', 'M46.01', 'M50.223', 'S12.600A', 'M54.06',
'M10.272', 'M12.571', 'M1A.4521', 'M86.451', 'M79.603', 'S22.031S', 'S24.132A', 'M46.94', 'M87.180',
'G44.301', 'M53.3', 'S32.048S', 'S34.114A', 'S32.2XXG', 'G43.829', 'M02.331', 'M25.551', 'M70.22',
'S22.000G', 'S22.41XG', 'M06.4', 'S12.201G', 'S14.133S', 'S12.231B', 'S12.54XD', 'S12.690S',
'M02.262', 'M02.321', 'M1A.0221', 'M1A.4311', 'M90.862', 'M70.41', 'M05.50', 'M99.36', 'M51.25',
'S22.001K', 'S24.113A', 'M49.81', 'M49.87', 'S12.040G', 'S12.291G', 'S12.331D', 'S14.127D',
'S12.631S', 'G90.512', 'M54.42', 'S32.058A', 'M10.049', 'M12.569', 'M67.341', 'G57.31', 'S34.139A',
'S32.2XXK', 'S22.042D', 'S12.030A', 'M60.9', 'M18.10', 'M01.X22', 'M01.X72', 'M1A.4790', 'G54.0',
'M05.521', 'M94.0', 'S22.039G', 'S22.049K', 'M05.422', 'S12.030B', 'S12.040B', 'S12.490B', 'G57.72',
'G52.1', 'M19.039', 'A18.01', 'S22.028G', 'S22.038D', 'S22.050B', 'S12.01XK', 'S14.131S',
'S12.14XD', 'S12.24XS', 'S12.391A', 'G44.039', 'M51.37', 'S32.131G', 'M02.319', 'M10.229',
'M10.239', 'M86.412', 'M75.102', 'S44.01XD', 'S22.010G', 'S22.041S', 'L93.2', 'M06.329', 'S14.114A',
'S12.330B', 'S12.650S', 'M99.53', 'S34.122A', 'S34.104D', 'M1A.2120', 'M25.532', 'M86.8X5',
'M70.21', 'B02.23', 'M05.772', 'M06.359', 'M49.88', 'M87.121', 'M50.122', 'S12.100D', 'S14.102S',
'S12.120B', 'S12.121B', 'M60.879', 'S32.022S', 'M02.869', 'M10.29', 'M1A.08X1', 'M65.112', 'M67.38',
'M77.51', 'M79.604', 'S22.078G', 'S24.103A', 'S22.21XA', 'M05.759', 'S12.330A', 'S12.351S',

'G90.523', 'S32.019B', 'M02.151', 'M02.161', 'M1A.4211', 'M46.24', 'M86.632', 'M89.632', 'M79.659', 'S54.02XS', 'S32.009B', 'M05.071', 'M06.032', 'M12.051', 'J34.0', 'M90.512', 'S12.300A', 'S14.152D', 'S12.131A', 'M26.631', 'M62.49', 'M02.279', 'M02.28', 'M1A.0490', 'M86.9', 'S24.103D', 'M32.0', 'M87.829', 'M50.021', 'S14.103D', 'S13.4XXD', 'S32.022D', 'S32.139B', 'G43.009', 'M00.179', 'M02.859', 'M1A.2520', 'M77.00', 'S24.153S', 'M05.272', 'M12.022', 'M46.84', 'S12.14XG', 'S12.491D', 'S34.114D', 'M02.861', 'M02.169', 'M1A.0110', 'M86.612', 'M76.71', 'G57.41', 'S12.111A', 'S12.501G', 'S32.112A', 'M62.421', 'M19.09', 'M02.261', 'S22.009S', 'M06.312', 'M06.871', 'M87.30', 'S12.151G', 'S12.430A', 'M60.872', 'S32.040B', 'G43.511', 'M1A.3390', 'M86.512', 'I70.222', 'I70.233', 'M65.859', 'M25.759', 'M25.775', 'M05.571', 'S22.022G', 'M06.0A', 'S14.133A', 'S12.251S', 'S12.631B', 'G90.522', 'M60.869', 'S32.032G', 'S32.110B', 'M00.272', 'M1A.3411', 'M1A.4610', 'M86.8X2', 'M99.26', 'S22.031D', 'M06.829', 'S14.104A', 'S12.390S', 'S14.125A', 'G44.85', 'S34.124A', 'S32.111A', 'S32.111D', 'M25.561', 'I70.229', 'S22.32XB', 'M05.069', 'S12.101A', 'S12.431K', 'S32.008D', 'M00.831', 'M02.38', 'M1A.08X0', 'M1A.3790', 'M86.539', 'S22.002S', 'M05.339', 'M05.461', 'S12.54XA', 'S32.119D', 'G43.701', 'M00.84', 'M00.162', 'M1A.0621', 'M1A.2591', 'M25.521', 'M25.531', 'M86.432', 'M86.561', 'S12.030D', 'S14.101D', 'S12.111K', 'S12.550S', 'G44.011', 'G43.C1', 'M00.231', 'M00.249', 'M1A.2191', 'M1A.2320', 'M1A.3121', 'M1A.48X0', 'M86.629', 'S54.00XA', 'S22.022S', 'M06.251', 'S12.000K', 'S12.300K', 'S12.400S', 'S32.009K', 'S32.018D', 'S32.041B', 'S32.15XG', 'M1A.28X1', 'M1A.4591', 'M79.2', 'S22.070A', 'S22.20XK', 'M05.349', 'S12.091G', 'S12.430G', 'S12.601K', 'S14.135A', 'S12.550A', 'M26.69', 'S32.019D', 'S32.028B', 'S32.039G', 'M00.879', 'M1A.2791', 'M1A.4121', 'M1A.4391', 'M86.69', 'M90.819', 'M79.652', 'S22.008K', 'S22.051G', 'S24.102A', 'S24.102S', 'S22.39XG', 'S14.103S', 'S32.029K', 'M19.279', 'M00.822', 'M1A.2421', 'M86.341', 'M65.169', 'M77.40', 'S44.02XD', 'S22.068B', 'S12.040D', 'G44.051', 'S34.129S', 'S32.131B', 'M00.049', 'M1A.0590', 'M86.40', 'M86.652', 'S22.032G', 'S32.008B', 'S32.020A', 'G43.B0', 'M10.172', 'M1A.2711', 'M83.2', 'S22.029A', 'S22.49XG', 'S22.49XK', 'M06.832', 'M46.86', 'M54.2', 'S12.430S', 'S12.9XXS', 'M53.86', 'S32.130B', 'S32.130S', 'M01.X0', 'M1A.0211', 'M86.452', 'M65.122', 'B02.22', 'S22.052D', 'S22.39XA', 'K62.89', 'S32.000S', 'M02.89', 'M86.449', 'M86.562', 'M90.821', 'M70.11', 'M79.671', 'M99.76', 'S22.072D', 'S24.151D', 'M05.279', 'M05.419', 'S12.190A', 'S12.290G', 'S32.021D', 'S32.14XB', 'M00.28', 'M10.219', 'M65.159', 'M79.602', 'G64', 'M99.28', 'M54.14', 'M06.232', 'M87.9', 'S14.111D', 'S12.14XK', 'R10.2', 'M62.449', 'M02.369', 'M02.379', 'M70.72', 'M79.629', 'S54.01XA', 'G57.40', 'G57.61', 'S22.050A', 'S22.051K', 'S22.068D', 'S22.43XD', 'M05.672', 'M87.10', 'M46.41', 'S16.1XXS', 'M99.33', 'M1A.2321', 'M86.511', 'I70.268', 'M75.31', 'M25.774', 'G62.2', 'M05.511', 'S24.109A', 'M05.562', 'S14.135S', 'G44.309', 'M51.17', 'M19.012', 'M1A.2311', 'M1A.3690', 'M46.20', 'M86.611', 'M76.50', 'M51.35', 'M05.662', 'S12.090G', 'S14.102D', 'S12.121K', 'S14.105D', 'M60.819', 'S32.008S', 'S34.131A', 'M02.362', 'M10.212', 'M1A.0711', 'M1A.3721', 'M76.32', 'M25.771', 'S44.01XS', 'G57.12', 'M99.64', 'S22.000B', 'M05.352', 'M06.08', 'M46.55', 'M50.01', 'M50.20', 'S12.550D', 'S12.630G', 'G57.71', 'S34.109D', 'S32.14XK', 'M19.241', 'M00.819', 'M00.112', 'M10.10', 'M89.641', 'M96.1', 'S22.020B', 'S24.151S', 'N94.818', 'G43.B1', 'M00.021', 'M1A.0790', 'M1A.2210', 'M65.132', 'M99.42', 'S22.041B', 'S22.048G', 'S22.061A', 'M05.371', 'S12.041B', 'S14.151S', 'S12.501D', 'S12.631D', 'M26.611', 'G44.099', 'M43.27', 'S32.012D', 'S32.028S', 'S32.040D', 'M02.239', 'M02.241', 'M10.042', 'M1A.0321', 'M1A.3521', 'M86.321', 'M86.522', 'M86.441', 'M86.669', 'M90.831', 'M76.821', 'M77.52', 'G61.9', 'G63', 'G65.2', 'S32.2XXD', 'S22.002K', 'S22.019K', 'M06.331', 'M87.20', 'S14.125S', 'G43.409', 'M25.559', 'M77.41', 'M77.8', 'M06.879', 'M32.19', 'S12.000A', 'S14.155A', 'S12.551B', 'S34.105S', 'M12.559', 'I70.245', 'M67.379', 'M25.739', 'M79.642', 'S32.032S', 'M00.161', 'M02.251', 'M12.579', 'M1A.2621', 'M67.361', 'M25.742', 'S22.080G', 'S22.32XS', 'L40.54', 'S12.231S', 'S12.651K', 'G90.50', 'M00.131', 'I70.25', 'I70.244', 'I73.9', 'M65.869', 'S34.109S', 'M51.85', 'S22.019G', 'S22.080K', 'S24.104A', 'S22.21XD', 'S22.41XD', 'M06.069', 'M06.39', 'M06.869', 'M46.81', 'S12.000B', 'S12.001B', 'S32.10XB', 'M19.072', 'M1A.2611', 'M89.672', 'M05.721', 'S12.14XB', 'S12.24XK', 'M26.639', 'M51.26', 'M54.31', 'M00.149', 'M02.219', 'M02.342', 'M02.351', 'M1A.20X1', 'S22.062D', 'S22.31XK', 'M05.652', 'M05.679', 'S12.201A', 'S12.112S', 'M26.649', 'S32.020B', 'S32.120B', 'S32.122B', 'M62.472', 'M1A.4690', 'M70.20', 'M75.51', 'M51.36', 'M54.16', 'M54.6', 'S22.051S', 'S22.22XK', 'M05.50', 'M46.96', 'S12.090A', 'S12.101G', 'S12.391G', 'S12.590S', 'M54.07', 'M00.169', 'M1A.39X0', 'M86.532', 'M67.329', 'G57.92',

'S22.009A', 'S22.032S', 'S22.062B', 'S22.081S', 'M50.923', 'S12.301B', 'S12.591G', 'G44.021', 'R51', 'M48.061', 'S34.139A', 'M17.30', 'M02.312', 'M75.82', 'S22.011S', 'M05.739', 'M05.79', 'S12.391K', 'S12.651S', 'M51.87', 'S32.021K', 'S32.110D', 'S32.2XXK', 'M19.041', 'M18.0', 'M00.821', 'M00.039', 'M77.11', 'S22.009G', 'M05.521', 'M06.352', 'S12.01XS', 'S12.120K', 'S12.150A', 'S12.44XG', 'S32.011D', 'S32.129S', 'M00.242', 'M86.39', 'M89.60', 'S22.042G', 'S24.111S', 'M05.431', 'M30.0', 'S13.8XXA', 'S32.018S', 'M1A.4410', 'M76.812', 'M76.819', 'G54.8', 'M99.47', 'S22.019D', 'M05.259', 'M05.452', 'M05.839', 'M12.032', 'M12.062', 'S12.001A', 'S14.136A', 'G44.89', 'S34.115S', 'S32.110A', 'M02.122', 'M1A.4620', 'M1A.49X0', 'M99.39', 'S22.24XD', 'M05.242', 'M31.7', 'M87.353', 'M87.174', 'S14.153D', 'S12.290D', 'G89.4', 'M16.30', 'M00.139', 'M01.X42', 'M1A.0320', 'M1A.3511', 'M86.8X3', 'S22.068A', 'S22.070K', 'S22.24XS', 'M05.362', 'M87.252', 'M50.33', 'S12.530S', 'S12.631G', 'M53.2X7', 'M54.41', 'S32.028G', 'S32.030A', 'M1A.3420', 'M89.622', 'M90.822', 'M77.20', 'M05.011', 'M12.059', 'S12.120A', 'S12.651B', 'S32.130D', 'M76.40', 'M99.72', 'S22.052K', 'S24.133S', 'M05.511', 'M34.0', 'M50.020', 'M50.81', 'M51.27', 'S32.008A', 'S34.105D', 'M54.09', 'M02.141', 'M1A.2420', 'M1A.4520', 'I70.235', 'M66.812', 'M79.669', 'S64.00XS', 'S22.001B', 'S22.22XS', 'M05.221', 'M06.029', 'S12.01XB', 'S12.501S', 'S14.115S', 'M00.861', 'M86.369', 'M89.611', 'M90.841', 'M65.822', 'S22.028B', 'S22.089D', 'S32.038A', 'S34.101D', 'M1A.0111', 'M89.661', 'M34.83', 'M51.14', 'S22.060B', 'S22.082A', 'M05.251', 'M87.851', 'M50.820', 'S12.301G', 'S12.290A', 'S12.291B', 'S14.107D', 'G44.209', 'S32.120A', 'S32.122G', 'M75.21', 'M79.641', 'S22.011A', 'S22.018D', 'M05.519', 'S12.34XK', 'S13.8XXS', 'M26.623', 'S34.109S', 'M02.222', 'M12.519', 'M1A.2721', 'M1A.4510', 'I70.223', 'M65.149', 'M76.02', 'M79.622', 'G56.93', 'S22.021G', 'M05.9', 'G57.73', 'S32.121G', 'M1A.09X1', 'M46.27', 'M76.9', 'M05.531', 'M05.542', 'S22.088G', 'M05.869', 'M05.879', 'S12.191S', 'S12.631K', 'S13.4XXA', 'S32.001S', 'S32.15XK', 'G43.A1', 'M02.841', 'M12.522', 'M89.652', 'M67.319', 'G57.10', 'S22.002G', 'M05.239', 'M49.85', 'S12.112G', 'S12.150D', 'S12.151K', 'S12.551G', 'M00.121', 'M1A.2221', 'M76.899', 'S32.009G', 'S22.011B', 'S22.021D', 'M05.451', 'M06.812', 'M12.021', 'S12.02XB', 'M60.829', 'S32.009S', 'S32.051D', 'M00.80', 'M02.819', 'M02.232', 'S34.139S', 'S22.079A', 'M05.021', 'S12.550G', 'S12.590A', 'N94.810', 'M96.1', 'G57.32', 'S24.111A', 'M06.049', 'S12.101S', 'S12.391S', 'S12.451S', 'M60.80', 'S32.039D', 'S34.112S', 'S32.110G', 'S32.19XG', 'M62.429', 'M10.222', 'M12.511', 'M1A.0520', 'M86.8X0', 'M75.112', 'S64.02XA', 'S22.019S', 'S22.040B', 'S22.43XK', 'L40.51', 'S14.124A', 'S12.121S', 'S14.116D', 'S32.032K', 'M16.12', 'M10.139', 'M1A.0491', 'A18.03', 'G60.8', 'M54.17', 'S22.058D', 'S22.059G', 'S22.069A', 'S22.069B', 'S22.072K', 'L93.1', 'M05.60', 'M05.651', 'M06.811', 'S12.500K', 'M00.211', 'M10.221', 'M1A.0511', 'M70.51', 'M79.673', 'S22.089A', 'S24.133A', 'M87.051', 'S12.201B', 'S14.137S', 'M00.122', 'M00.222', 'M02.852', 'M10.112', 'M75.20', 'M76.61', 'S22.070G', 'L40.52', 'M05.412', 'M05.722', 'S14.122S', 'S14.132S', 'S12.110K', 'S12.151A', 'S12.630S', 'S34.123A', 'S34.123D', 'M19.179', 'M1A.0791', 'M26.622', 'M90.879', 'I70.234', 'M05.532', 'S34.109A', 'S32.2XXB', 'M87.075', 'S14.101A', 'S32.001B', 'M02.259', 'M02.271', 'M10.011', 'M25.731', 'M99.27', 'S32.2XXG', 'M05.769', 'M06.071', 'S12.401G', 'S12.490D', 'S32.020D', 'S32.052B', 'S34.113D', 'S32.10XS', 'S32.129D', 'S32.132A', 'M19.232', 'M19.242', 'M01.X21', 'M11.10', 'M25.512', 'M65.10', 'G57.00', 'G58.9', 'M99.78', 'S22.028S', 'M06.841', 'M46.52', 'M46.97', 'S12.02XS', 'S12.14XA', 'S12.191G', 'S12.391D', 'S12.490G', 'S12.591A', 'S32.002S', 'G43.709', 'G43.909', 'M18.4', 'M46.46', 'S32.009A', 'S32.018A', 'S32.040K', 'M36.2', 'I70.263', 'M67.30', 'M48.08', 'M06.821', 'M34.9', 'M90.519', 'M90.559', 'S14.157D', 'S32.001G', 'S32.030S', 'M17.11', 'M00.229', 'M02.162', 'M12.551', 'M70.62', 'M77.01', 'M79.645', 'S22.060S', 'M05.812', 'S14.151D', 'S12.251A', 'S12.291A', 'S12.600D', 'M60.832', 'S34.121D', 'G43.819', 'M10.062', 'M1A.4621', 'M86.319', 'M48.05', 'S22.068G', 'M05.062', 'M05.779', 'M34.82', 'M87.019', 'M87.859', 'S12.351G', 'S14.156S', 'S12.551S', 'M60.861', 'S32.009D', 'G43.C0', 'M02.871', 'M10.09', 'M90.861', 'M67.349', 'S22.040S', 'M05.659', 'M05.841', 'M06.88', 'M50.823', 'S14.103A', 'S12.650A', 'G44.041', 'S32.011K', 'S32.122A', 'M02.851', 'M01.X8', 'M10.022', 'M12.549', 'M79.651', 'M05.30', 'M05.631', 'M06.019', 'S12.151B', 'S12.331S', 'S12.44XK', 'G43.411', 'G43.519', 'M00.18', 'M1A.9XX1', 'M1A.2790', 'M86.8X1', 'S64.01XD', 'E10.42', 'S32.10XA', 'S22.050K', 'S22.49XD', 'M46.89', 'S12.000D', 'S14.112S', 'S14.113S', 'S14.124S', 'S32.16XK', 'M00.859', 'M1A.0120', 'M1A.3710', 'M86.461', 'F44.6', 'M05.59', 'S22.059D', 'S22.072S', 'M32.12', 'S12.110D', 'M62.40', 'M83.3', 'M67.322', 'M70.61', 'M54.10', 'M46.48', 'S34.109D', 'S22.008B', 'S22.018A', 'S22.061K',

'S12.100A', 'S14.131A', 'M53.2X8', 'S32.15XB', 'M19.142', 'M00.221', 'M1A.0290', 'M86.8X7', 'G57.43', 'M51.24', 'M46.99', 'S12.300D', 'S12.600B', 'S32.17XS', 'M10.149', 'M1A.0691', 'M76.72', 'G56.92', 'S22.028D', 'S22.041K', 'S22.070S', 'S22.082B', 'S22.20XB', 'M05.359', 'M05.472', 'S12.251K', 'M02.172', 'M10.129', 'M1A.0291', 'M76.20', 'M75.101', 'G57.52', 'G57.53', 'M05.539', 'S32.10XD', 'S22.089K', 'S24.114A', 'M05.641', 'S12.001K', 'S12.250K', 'S12.601S', 'S32.049B', 'M62.830', 'M00.152', 'M1A.0311', 'M46.07', 'S24.109S', 'S22.018G', 'S22.088B', 'M50.21', 'S12.031G', 'M00.159', 'M89.68', 'M67.331', 'M79.609', 'G56.81', 'G58.0', 'S22.010S', 'M05.871', 'M12.011', 'S12.000G', 'M26.613', 'S32.002D', 'S32.032B', 'S32.049A', 'S32.112S', 'S34.139D', 'G43.801', 'M00.051', 'M01.X29', 'M02.152', 'M10.119', 'M10.262', 'M1A.40X0', 'M25.511', 'G56.21', 'M99.49', 'M05.39', 'S14.111S', 'S12.120D', 'S12.191D', 'S12.250A', 'S12.351A', 'S12.431A', 'S12.64XD', 'S32.029A', 'S32.031D', 'M16.50', 'M10.142', 'M25.542', 'M86.311', 'M86.431', 'M66.819', 'S22.23XB', 'M05.669', 'M05.411', 'M06.052', 'S12.290S', 'S12.390G', 'S14.115A', 'S14.127A', 'S12.531S', 'S32.029B', 'S32.030K', 'S32.049G', 'M00.20', 'M1A.0191', 'M1A.3490', 'M70.71', 'S64.00XD', 'S22.008D', 'S22.051B', 'M05.271', 'S14.104D', 'S12.331G', 'S12.450B', 'S12.691B', 'M60.859', 'S32.052G', 'S32.121A', 'M19.141', 'M00.839', 'M12.521', 'M86.442', 'M65.852', 'M76.22', 'M25.776', 'S22.008G', 'M06.229', 'S32.112B', 'M62.419', 'M16.51', 'M19.219', 'M19.139', 'M00.041', 'M25.749', 'M66.811', 'M77.31', 'M05.59', 'M06.29', 'M49.80', 'M50.31', 'M50.80', 'S12.650G', 'S32.120G', 'S32.2XXB', 'M1A.4320', 'M86.359', 'G57.90', 'M05.552', 'M46.44', 'S24.132D', 'M05.342', 'M06.231', 'M87.052', 'M87.152', 'S12.001D', 'S12.091D', 'S14.107A', 'G44.059', 'M17.5', 'M10.021', 'M1A.2490', 'M1A.3110', 'M48.04', 'M06.079', 'S12.01XD', 'S12.500A', 'S16.1XXA', 'S16.1XXD', 'M62.439', 'M1A.3290', 'M1A.4691', 'M86.419', 'M77.21', 'M46.05', 'S32.10XG', 'S22.028K', 'S22.089S', 'L40.53', 'M12.049', 'S12.231D', 'S32.028D', 'S32.039S', 'S32.111B', 'M02.339', 'M83.8', 'G56.80', 'S22.030B', 'S22.071S', 'M50.321', 'S14.101S', 'S12.401S', 'S14.136D', 'S32.051S', 'S34.103A', 'M54.03', 'M00.059', 'M10.162', 'S44.00XA', 'G62.0', 'S22.071A', 'S14.121S', 'S12.120S', 'M51.06', 'M02.812', 'M10.072', 'M1A.0720', 'M89.631', 'M76.41', 'M46.57', 'S12.430B', 'S32.022G', 'S32.119G', 'M62.442', 'M19.121', 'M00.019', 'M00.269', 'M02.349', 'M10.122', 'M10.259', 'S54.02XD', 'M46.02', 'S22.028A', 'S22.078S', 'S12.120G', 'G90.521', 'M26.633', 'M18.51', 'M00.151', 'M10.131', 'I70.249', 'M70.52', 'S22.031G', 'M05.559', 'M06.259', 'S32.031A', 'M16.11', 'M19.119', 'M65.80', 'S64.01XA', 'S22.009B', 'S24.152S', 'M05.542', 'M05.742', 'M06.342', 'S12.590B', 'S32.019G', 'M19.211', 'M1A.2710', 'M05.029', 'M06.379', 'M46.56', 'S12.041S', 'S12.231G', 'S12.44XS', 'S14.127S', 'M60.871', 'S32.019K', 'S32.10XK', 'G43.911', 'M19.122', 'M25.572', 'M86.322', 'M90.88', 'M25.773', 'M99.38', 'S22.012B', 'S22.032K', 'S22.048K', 'S12.02XG', 'S32.031S', 'M12.562', 'S32.10XB', 'M05.661', 'M05.822', 'M06.012', 'M06.861', 'M12.00', 'R09.81', 'M87.850', 'S12.100K', 'S12.200B', 'S12.231K', 'S12.600K', 'S12.650B', 'S32.022B', 'S32.048G', 'S32.059K', 'S32.131D', 'M17.12', 'M1A.00X1', 'M1A.3190', 'M77.22', 'M75.30', 'M76.892', 'S22.031A', 'M05.831', 'M05.89', 'M06.20', 'M06.272', 'S12.490S', 'S12.9XXA', 'M15.3', 'M89.679', 'S22.080S', 'S22.22XB', 'S22.31XA', 'M05.00', 'M05.712', 'M87.129', 'M54.81', 'S12.301K', 'S12.630B', 'S12.64XS', 'S32.120D', 'S32.139K', 'M00.079', 'M1A.3620', 'M86.339', 'I70.238', 'I73.1', 'M76.42', 'M79.644', 'M05.249', 'M46.92', 'M87.059', 'S12.250D', 'S34.111D', 'S32.119A', 'S32.121D', 'M00.061', 'M89.69', 'S22.079B', 'M05.231', 'M05.459', 'M05.471', 'M06.261', 'M87.852', 'S12.191A', 'G89.29', 'M60.862', 'S32.039K', 'S32.132D', 'S34.132A', 'G43.509', 'M00.031', 'M46.26', 'M86.621', 'M76.891', 'M79.661', 'G62.89', 'S12.9XXD', 'M46.45', 'S22.31XD', 'S22.41XB', 'M06.021', 'S12.130B', 'S14.126A', 'S12.530D', 'M26.602', 'S32.011G', 'M00.852', 'M02.832', 'M46.25', 'S22.002A', 'S22.062K', 'S12.131G', 'M02.132', 'S14.121A', 'M79.7', 'M54.04', 'M01.X12', 'M79.643', 'R25.2', 'S22.081A', 'S24.104S', 'S24.133D', 'S22.39XD', 'M05.012', 'M05.612', 'M05.429', 'M06.831', 'M06.872', 'M54.30', 'M00.069', 'M1A.3610', 'M75.22', 'S24.152A', 'M05.762', 'M06.072', 'M50.93', 'S12.100S', 'S12.301S', 'S14.113D', 'S12.291S', 'S12.451K', 'M00.141', 'M00.261', 'M00.279', 'M02.821', 'M02.18', 'M86.422', 'M67.311', 'M79.672', 'S24.131D', 'S24.132S', 'L40.50', 'M05.022', 'M50.123', 'S12.250S', 'S12.451A', 'S32.050G', 'M19.042', 'M1A.2521', 'M1A.3791', 'E09.40', 'S22.018B', 'S22.030G', 'S22.041G', 'S12.491B', 'M19.271', 'M12.58', 'M1A.3111', 'M46.28', 'M89.662', 'M79.639', 'G57.42', 'S22.012D', 'S22.088S', 'M12.061', 'S14.111A', 'S12.24XA', 'S12.330K', 'S12.450A', 'S12.491A', 'S32.019A', 'S32.059G', 'M19.231', 'M00.172', 'M02.249', 'M1A.3120', 'M1A.3410', 'G61.89', 'M99.48', 'S22.009D', 'S24.113D', 'S24.154A', 'M90.551',

'S12.02XD', 'S14.131D', 'S14.106A', 'M99.43', 'S32.038B', 'M16.7', 'M86.662', 'M86.371', 'M65.829',
'S22.058A', 'S24.154S', 'M87.022', 'S12.290K', 'S32.001D', 'S32.14XD', 'M25.579', 'S24.153A',
'M34.81', 'M53.82', 'S12.391B', 'S32.139D', 'G43.831', 'M12.561', 'M1A.3391', 'M25.539', 'M75.01',
'M06.219', 'S12.101K', 'S14.154A', 'S12.350B', 'S34.113S', 'S32.122S', 'M19.011', 'M86.661',
'M86.471', 'M89.612', 'M65.172', 'M76.31', 'F44.4', 'M05.861', 'M06.842', 'M46.82', 'A18.01',
'M87.375', 'S12.041K', 'S12.600G', 'G43.619', 'M19.022', 'M10.061', 'M1A.0610', 'M1A.0710',
'M86.362', 'M65.119', 'M65.162', 'M50.023', 'S12.000S', 'S12.02XA', 'S13.8XXD', 'M26.642',
'M10.242', 'M1A.2190', 'M83.4', 'M86.659', 'M67.39', 'M77.42', 'M99.22', 'S24.131A', 'M06.341',
'M12.08', 'M53.81', 'S12.531K', 'G44.031', 'S34.122D', 'M62.48', 'M02.322', 'T14.8XXA', 'M06.9',
'M05.819', 'M06.362', 'M60.821', 'S34.122S', 'M62.422', 'M00.022', 'M00.071', 'M25.552', 'M86.60',
'M75.80', 'M79.601', 'S54.00XD', 'S12.121A', 'S12.291K', 'S12.330D', 'S14.157S', 'S32.129K',
'M62.479', 'M62.831', 'M00.032', 'M00.9', 'M02.159', 'M1A.3221', 'M1A.4421', 'M67.339', 'S22.010K',
'S22.012K', 'S22.020G', 'S32.050A', 'S34.119A', 'S32.110K', 'M00.872', 'M00.09', 'M00.29',
'M02.112', 'M1A.0421', 'M1A.4290', 'M76.829', 'G56.82', 'S22.072G', 'S22.20XG', 'S12.500S',
'S12.551A', 'S32.032A', 'S34.103S', 'S34.131D', 'M15.2', 'M02.811', 'M10.069', 'M86.572', 'M76.12',
'M79.674', 'S22.018S', 'S22.21XB', 'M05.572', 'M05.731', 'M06.89', 'M46.58', 'M50.121', 'S12.112K',
'S32.030G', 'S32.058K', 'S34.121A', 'M18.32', 'M1A.3520', 'S64.00XA', 'S14.109S', 'S22.009D',
'S22.029B', 'S22.059A', 'S24.112D', 'S24.131S', 'M05.49', 'M06.222', 'M35.3', 'J34.89', 'S12.091B',
'S12.24XD', 'S32.130G', 'M15.1', 'M19.021', 'M02.149', 'M12.512', 'M1A.40X1', 'M1A.0411',
'M67.371', 'M05.562', 'S14.109A', 'S22.072A', 'M05.761', 'M06.822', 'M87.256', 'S12.230D',
'S12.250B', 'S32.002B', 'S34.112A', 'I87.2', 'M79.605', 'F45.41', 'S22.011K', 'M06.862', 'M46.54',
'M87.012', 'S14.151A', 'S12.501K', 'G90.519', 'G44.1', 'S34.104A', 'S32.14XS', 'M16.32', 'M1A.4590',
'M86.411', 'M67.332', 'M99.75', 'S22.001G', 'S22.012S', 'M06.322', 'M90.552', 'S14.134A',
'S34.115D', 'S32.111K', 'M00.171', 'M86.30', 'M86.49', 'M89.659', 'S22.010D', 'S22.42XG',
'S12.191K', 'S12.351B', 'S14.126D', 'S12.591S', 'M60.852', 'G43.809', 'M00.132', 'M05.619', 'M46.1',
'M00.10', 'M1A.0220', 'M25.519', 'M86.462', 'S22.071B', 'S14.121D', 'S12.431S', 'G90.59',
'S32.19XK', 'S34.139S', 'M86.342', 'M67.352', 'G57.13', 'M05.561', 'M99.67', 'S22.009K', 'S22.071G',
'S24.134S', 'S14.123D', 'S12.230A', 'S32.058S', 'S32.17XA', 'M62.412', 'M10.252', 'M86.542',
'I70.269', 'S22.029K', 'S22.049A', 'S22.061B')
